# OLDER ADULTS’ PERCEPTION TOWARD DEPRESCRIBING INAPPROPRIATE MEDICATIONS

**DOI:** 10.1093/geroni/igae098.2841

**Published:** 2024-12-31

**Authors:** Mohammad Rababa, Ali Al Ghazo

**Affiliations:** Jordan University Of Science And Technology, Irbid, Irbid, Jordan; Jordan University Of Science And Technology, Irbid, Irbid, Jordan

## Abstract

**Introduction:**

Inappropriate prescription of medications is a problem of growing interest in geriatrics. Initiatives have been ongoing to evaluate interventions targeting the hazards of inappropriate prescription of medications; Deprescribing is one of these initiatives. However, no current study has examined medicine-related perceptions toward deprescribing inappropriate medications among older adults.

**Methods:**

A convenience sample of older adults who regularly visited the outpatient clinics during July 2023 for regular checkups were recruited. Data were collected by a graduate nursing student from one pharmacy located in a public hospital, five days per week, via in-person interviews using the Patient’s Perceptions of Deprescribing survey.

**Results:**

The study found that older adults’ perceived interest in stopping medications, belief about medicine overuse, the unimportance of medicines, and medication concerns are significantly associated with a variety of sociodemographic (e.g., age), clinical (e.g., comorbidities), and PCP-related factors (e.g., knowledge of medications).

**Conclusion:**

Inappropriate prescription of medication is one of the most common polypharmacy-related issues among older adults. The current study reported its findings on medicine-related perception toward deprescribing inappropriate medications among older adults. Care providers should discuss the benefits of deprescribing inappropriate medications with their patients to prevent the side effects associated with long-term unnecessary use. The long-term use of inappropriate medications by older adults should be carefully evaluated. Future studies on the effectiveness of an evidence-based deprescribing protocol on minimizing the clinical side effects associated with inappropriate prescription of medications among older adults are recommended.

